# Supplementary material for: Molecular typing and epidemiology profiles of human adenovirus infection among hospitalized patients with severe acute respiratory infection in Huzhou, China
Source: PLoS One. 2022 Apr 21;17(4):e0265987. doi: 10.1371/journal.pone.0265987 (PMC9022850; doi:10.1371/journal.pone.0265987)

PLOS ONE

Molecular Typing and Epidemiology Profiles of Human Adenovirus Infection among Hospitalized Patients with Severe Acute Respiratory Infection in Huzhou, China

--Manuscript Draft--

**Manuscript Number:**	PONE-D-21-05692
**Article Type:**	Research Article
**Full Title:**	Molecular Typing and Epidemiology Profiles of Human Adenovirus Infection among Hospitalized Patients with Severe Acute Respiratory Infection in Huzhou, China
**Short Title:**	Human Adenovirus Infection in Hospitalized Patients in China
**Corresponding Author:**	Lei Ji<br>Huzhou Center for Disease Control and Prevention<br>Huzhou, CHINA
**Keywords:**	Human adenovirus; Respiratory tract infection; Epidemiology
**Abstract:**	Background: Severe acute respiratory infections (SARI) threaten human health and cause a large number of hospitalized patients every year. However, as one of the most common pathogen that cause acute respiratory tract infection, the molecular epidemiological information relating to HAdV among patients with SARI is limited. Here, we evaluate the epidemiological and molecular characteristics of HAdV infections among hospitalized patients with SARI from January 2017 to December 2019 in Huzhou, China.<br>Methods: From January 2017 to December 2019, a total of 657 nasopharyngeal swabs collected from inpatients with SARI were screened for HAdV and other common respiratory viruses by multiplex real-time PCR. All samples that tested positive for HAdV were further typed by sequencing partial sequences of hexon gene. Genotypes of HAdV were confirmed by phylogenetic analysis. Epidemiological data were analyzed using Microsoft Excel 2010 and service solutions (SPSS) 21.0 software.<br>Results: 251 (38.20%) samples were positive for at least one respiratory virus. HAdV was the second common viral pathogen detected, with a detection rate of 7.08%. Infection with HAdV was found in all age groups tested(0 ~ , 2 ~ , 5 ~ ,15 ~ , 50 ~ , 65 ~). Children under 15 years old accounted for 84.62% (44/52) of the infections. Higher activity of HAdV infection could be seen in spring-early autumn season. 7 different types of HAdV belonging to 4 species (HAdV-A, B, C, E) were identified in hospitalized SARI cases, with HAdV-B3 as the most prevalent HAdV types, followed by HAdV-B7 and HAdV-E4. HAdV-B3 was the most frequently detected genotype in 2017and 2019, accounting for 75.00% (9/12) and 63.64(7/11) of typed HAdV infections in 2017 and 2019 respectively. No predominant strain was responsible for HAdV infections in 2018, although HAdV-B7 (28.57%, 2/7) and HAdV-C1 (28.57%,2/7)were the major causative genotypes.<br>Conclusions: This study revealed the prevalence and the molecular epidemiological characteristics of HAdV infections among hospitalized patients with SARI in Huzhou from January 2017 to December 2019. The HAdV prevalence is related to age and season. As the most prevalent HAdV types, HAdV-B3 was co-circulating with other types and presented an alternate prevalence pattern.
**Order of Authors:**	Deshun Xu
	Liping Chen
	Xiaofang Wu
	Lei Ji
**Additional Information:**	
**Question**	**Response**
**Financial Disclosure**<br><br>Enter a financial disclosure statement that describes the sources of funding for the	The author(s) received no specific funding for this work

work included in this submission. Review the submission guidelines for detailed requirements. View published research articles from *PLOS ONE* for specific examples.<br><br>This statement is required for submission and **will appear in the published article** if the submission is accepted. Please make sure it is accurate.<br><br>**Unfunded studies**<br>Enter: *The author(s) received no specific funding for this work.*<br><br>**Funded studies**<br>Enter a statement with the following details:<br>• Initials of the authors who received each award<br>• Grant numbers awarded to each author<br>• The full name of each funder<br>• URL of each funder website<br>• Did the sponsors or funders play any role in the study design, data collection and analysis, decision to publish, or preparation of the manuscript?<br>• **NO** - Include this sentence at the end of your statement: *The funders had no role in study design, data collection and analysis, decision to publish, or preparation of the manuscript.*<br>• **YES** - Specify the role(s) played.<br><br>* typeset	
**Competing Interests**<br><br>Use the instructions below to enter a competing interest statement for this submission. On behalf of all authors, disclose any competing interests that could be perceived to bias this work—acknowledging all financial support and any other relevant financial or non-financial competing interests.<br><br>This statement is required for submission and **will appear in the published article** if the submission is accepted. Please make sure it is accurate and that any funding sources listed in your Funding Information	The authors have declared that no competing interests exist

later in the submission form are also declared in your Financial Disclosure statement.<br><br>View published research articles from *PLOS ONE* for specific examples.<br><br>**NO authors have competing interests**<br><br>Enter: *The authors have declared that no competing interests exist.*<br><br>**Authors with competing interests**<br><br>Enter competing interest details beginning with this statement:<br><br>*I have read the journal's policy and the authors of this manuscript have the following competing interests: [insert competing interests here]*<br><br>* typeset	
**Ethics Statement**<br><br>Enter an ethics statement for this submission. This statement is required if the study involved:<br><br>• Human participants<br>• Human specimens or tissue<br>• Vertebrate animals or cephalopods<br>• Vertebrate embryos or tissues<br>• Field research<br><br>Write "N/A" if the submission does not require an ethics statement.<br><br>General guidance is provided below. Consult the submission guidelines for detailed instructions. **Make sure that all information entered here is included in the Methods section of the manuscript.**	This study was part of the national SARI surveillance program and was approved by the human research ethics committee of Huzhou Center for Disease Control and Prevention. The only human materials used were nasopharyngeal swabs collected from patients for routine detection. Oral informed consents were obtained from each participant.

**Format for specific study types**<br><br>**Human Subject Research (involving human participants and/or tissue)**<br>• Give the name of the institutional review board or ethics committee that approved the study<br>• Include the approval number and/or a statement indicating approval of this research<br>• Indicate the form of consent obtained (written/oral) or the reason that consent was not obtained (e.g. the data were analyzed anonymously)<br><br>**Animal Research (involving vertebrate animals, embryos or tissues)**<br>• Provide the name of the Institutional Animal Care and Use Committee (IACUC) or other relevant ethics board that reviewed the study protocol, and indicate whether they approved this research or granted a formal waiver of ethical approval<br>• Include an approval number if one was obtained<br>• If the study involved *non-human primates*, add *additional details* about animal welfare and steps taken to ameliorate suffering<br>• If anesthesia, euthanasia, or any kind of animal sacrifice is part of the study, include briefly which substances and/or methods were applied<br><br>**Field Research**<br><br>Include the following details if this study involves the collection of plant, animal, or other materials from a natural setting:<br>• Field permit number<br>• Name of the institution or relevant body that granted permission	
**Data Availability**<br><br>Authors are required to make all data underlying the findings described fully available, without restriction, and from the time of publication. PLOS allows rare exceptions to address legal and ethical concerns. See the PLOS Data Policy and FAQ for detailed information.	Yes - all data are fully available without restriction

A Data Availability Statement describing where the data can be found is required at submission. Your answers to this question constitute the Data Availability Statement and **will be published in the article**, if accepted.<br><br>**Important:** Stating 'data available on request from the author' is not sufficient. If your data are only available upon request, select 'No' for the first question and explain your exceptional situation in the text box.<br><br>Do the authors confirm that all data underlying the findings described in their manuscript are fully available without restriction?	
**Describe where the data may be found in full sentences. If you are copying our sample text, replace any instances of XXX with the appropriate details.**<br><br>• If the data are **held or will be held in a public repository**, include URLs, accession numbers or DOIs. If this information will only be available after acceptance, indicate this by ticking the box below. For example: *All XXX files are available from the XXX database (accession number(s) XXX, XXX.*).<br>• If the data are all contained **within the manuscript and/or Supporting Information files**, enter the following: *All relevant data are within the manuscript and its Supporting Information files.*<br>• If neither of these applies but you are able to provide **details of access elsewhere**, with or without limitations, please do so. For example:<br><br>*Data cannot be shared publicly because of [XXX]. Data are available from the XXX Institutional Data Access / Ethics Committee (contact via XXX) for researchers who meet the criteria for access to confidential data.*<br><br>*The data underlying the results presented in the study are available from (include the name of the third party*	The sequences of the HAdV strains obtained in this study were deposited in the GenBank under the accession numbers MW594169-MW594198

and contact information or URL).<br>• This text is appropriate if the data are owned by a third party and authors do not have permission to share the data.<br><br>* typeset	
Additional data availability information:	

Powered by Editorial Manager® and ProduXion Manager® from Aries Systems Corporation

Manuscript

Click here to access/download;Manuscript;20210218Manuscript.docx

Title:

Molecular Typing and Epidemiology Profiles of Human Adenovirus Infection among Hospitalized Patients with Severe Acute Respiratory Infection in Huzhou, China

Short title:

Human Adenovirus Infection in Hospitalized Patients in China

Deshun Xu, Liping Chen, Xiaofang Wu, Lei Ji

Author affiliations:
Huzhou Center for Disease Control and Prevention, Huzhou, Zhejiang Province, China

Email address:
Lei Ji: jileichn@163.com
Deshun Xu: xds666092@126.com
Liping Chen: lipingchen1106@hotmail.com
Xiaofang Wu: xf980718@126.com

*Corresponding Author: Lei Ji
Email address: jileichn@163.com

Abstract

Background: Severe acute respiratory infections (SARI) threaten human health and cause a large number of hospitalized patients every year. However, as one of the most common pathogen that cause acute respiratory tract infection, the molecular epidemiological information relating to HAdV among patients with SARI is limited. Here, we evaluate the epidemiological and molecular characteristics of HAdV infections among hospitalized patients with SARI from January 2017 to December 2019 in Huzhou, China.

Methods: From January 2017 to December 2019, a total of 657 nasopharyngeal swabs collected from inpatients with SARI were screened for HAdV and other common respiratory viruses by multiplex real-time PCR. All samples that tested positive for HAdV were further typed by sequencing partial sequences of hexon gene. Genotypes of HAdV were confirmed by phylogenetic analysis. Epidemiological data were analyzed using Microsoft Excel 2010 and service solutions (SPSS) 21.0 software.

Results: 251 (38.20%) samples were positive for at least one respiratory virus. HAdV was the second common viral pathogen detected, with a detection rate of 7.08%. Infection with HAdV was found in all age groups tested(0～, 2～, 5～,15～, 50～, 65～). Children under 15 years old accounted for 84.62% (44/52) of the infections. Higher activity of HAdV infection could be seen in spring-early autumn season. 7 different types of HAdV belonging to 4 species (HAdV-A, B, C, E) were identified in hospitalized SARI cases, with HAdV-B3 as the most prevalent HAdV types, followed by HAdV-B7 and HAdV-E4. HAdV-B3 was the most frequently detected genotype in 2017and 2019, accounting for 75.00% (9/12) and 63.64(7/11) of typed HAdV infections in 2017 and 2019 respectively. No predominant strain was responsible for HAdV infections in 2018, although HAdV-B7 (28.57%, 2/7) and HAdV-C1 (28.57%,2/7)were the major causative genotypes.

Conclusions: This study revealed the prevalence and the molecular epidemiological characteristics of HAdV infections among hospitalized patients with SARI in Huzhou from January 2017 to December 2019. The HAdV prevalence is related to age and season. As the most prevalent HAdV types, HAdV-B3 was co-circulating with other types and presented an alternate prevalence pattern.

Keywords: Human adenovirus ; Respiratory tract infection ; Epidemiology

Background

Human adenoviruses (HAdVs) are non-enveloped, double-stranded DNA viruses belonging to the genus Mastadenovirus of the Adenoviridae family[1]. HAdVs have been recognized as pathogens that cause a broad spectrum of diseases, including respiratory illness, keratoconjunctivitis, gastroenteritis, cystitis and meningoencephalitis[2, 3]. They are associated with sporadic infection, as well as with community and institutional outbreaks. As a significant causative agent of respiratory tract illnesses, HAdV accounts for at least 5 to 10% of pediatric and 1 to 7% of adult respiratory tract infections (RTIs)[4, 5].

There are currently seven different HAdV species (HAdV-A through HAdV-G), and to date, 51 serotypes and over 70 genotypes have been identified based on serology, phylogenetic analyses and whole genomic sequencing (http://hadvwg.gmu.edu/). Different types of HAdVs display

different tissue tropisms that correlate with clinical manifestations of infection. The HAdV types most commonly associated with respiratory infection belong to HAdV species B (HAdV-3,HAdV-7, HAdV-11, HAdV-14, HAdV-21), HAdV species C (HAdV-C1, -C2, -C5, and -C6) and HAdV species E (HAdV-4)[3].

The predominant types of HAdV circulating at a given time differ among countries or regions and change over time. Replacement of dominant viruses by new strains may occur because transmission of novel strains between countries. During the last decade, outbreaks of respiratory tract infections caused by novel HAdV srains have occurred frequently in many countries including China[6, 7]. Therefore, clarifying the genotype of HAdV currently circulating is essential for epidemiological surveillance and a better understanding of the epidemic pattern of HAdV infection. At present, China has not yet established a national HAdV surveillance system. Although data about HAdV associated with respiratory infection in China can be found in several studies, most studies are performed with specific groups, especially for children[8-13]. There is a lack of epidemiological analyses of HAdV associated respiratory infection among patients in all age groups in China. The aim of this study was to evaluate the epidemiological and molecular characteristics of HAdV infections among hospitalized patients with severe acute respiratory infection (SARI) from January 2017 to December 2019 in Huzhou, a medium-sized city located in eastern China.

Materials and methods

Ethics statement

This study was part of the national SARI surveillance program and was approved by the human research ethics committee of Huzhou Center for Disease Control and Prevention. The only human materials used were nasopharyngeal swabs collected from patients for routine detection. Data records and collected clinical specimens were deidentified and anonymous. Oral informed consents were obtained from each participant.

Patients and specimens

During the influenza A H1N1 epidemic in 2009, a surveillance system for SARI was established to monitor influenza infection in these cases in china, As local SARI surveillance sentinel hospital, the First People's Hospital of Huzhou was responsible for sample collection from surveillance cases. The inclusion criteria for hospitalized SARI cases were as follows: the onset of the disease has a history of fever(> 38°C), accompanied by cough, and the onset does not exceed 10 days. Nasopharyngeal swabs were freshly collected and sent to Huzhou Center for Disease Control and Prevention for routine detection. All the specimens were stored at − 80 °C until further processing. Demographic and clinical data were obtained from the hospital's database.

Detection of HAdV and other common respiratory viruses

Total viral nucleic acids (DNA and RNA) were extracted from 200 μL of each specimen using TIANLONG Ex Viral DNA/RNA Kit (TIANLONG Biotech, Xi' an, China) according to the manufacturer's instructions. Multiplex real-time PCR kit (BioGerm, Shanghai, China) was used to detect HAdV and other common respiratory virus pathogens, including Human Influenza virus(HIFV), Human respiratory syncytial virus(HRSV), Human rhinovirus(HRV), Human bocavirus(HBOV), Human metapneumovirus(HMPV), Human Parainfluenza Virus(HPIV) type 1~4 and Human coronavirus (HCoV). The qPCR cycling program was as follows: 50 °C for 10

min, 95 °C for 5min, followed by 40 cycles of 95 °C for 10 s, and 55 °C for 40 s. Samples with a cycle threshold (Ct) < 35 were regarded as positive.

HAdV genotyping

HAdV-positive samples were further molecularly typed by nested PCR amplification and sequencing of HAdV hexon gene hyper-variable regions 1–6 (HVR1−6) as described previously[14]. Primer set AdhexF1 (nt 19135–19160;5′-TICTTTGACATICGIGGIGTICTIGA-3′) and AdhexR1 (nt 20009–20030;5′-CTGTCIACIGCCTGRTTCCACA-3′) were used for first-round amplification; a second-round PCR was performed using primer set AdhexF2 (nt 19165–19187; 5′-GGYCCYAGYTTYAARCCCTAYTC-3′) and AdhexR2 (nt 19960–19985; 5′-GGTTCTGTCICCCAGAGARTCIAGCA-3′) if insufficient DNA was amplified from the first reaction for sequencing. The PCR products were visualized by electrophoresis and sent to TaKaRa Biotechnology (Dalian, China) for further purication and sequencing.

Phylogenetic analysis

Partial nucleotide sequences of hexon gene obtained in this study were compared with the NCBI GenBank database (http://www.ncbi.nlm.nih.gov) by using online BLAST tools to preliminarily determine the genotype. Multiple sequence alignment and phylogenetic analysis were conducted using MEGA software version 6.06. The phylogenetic tree was generated using the neighbor-joining method and bootstrap analysis was performed with 1000 replications.

Statistical analysis

Epidemiological data were analyzed using Microsoft Excel 2010 and service solutions (SPSS) 21.0 software. Statistical differences were determined using the Chi-square test and P-values <0.05 were considered to represent a statistically significant difference.

Accession numbers

The partial hexon gene sequences obtained in this study have been deposited in GenBank under the accession numbers MW594169-MW594198.

Results

Characteristics of the SARI cases and the Viral infection profiles

From January 2017 to December 2019，a total of 657 specimens (191 in 2017, 204 in 2018 and 262 in 2019) were collected from inpatients with SARI during the study period~~. Among those SARI cases~~, 361 (54.95%) were male and 296 (45.05%) were female~~, the~~ age range was from 1 month to 86 years old with 590 (89.80%) cases ~~were children younger than 15 years old.~~

The viral infection profiles are shown in Table 1. Overall, 251 (38.20%) samples were positive for at least one respiratory virus, the detection rate of respiratory virus was 45.54% (87/191) in 2017, 36.27% (74/204) in 2018 and 34.35% (90/262) in 2019. During the study period, the most commonly detected viral pathogen in SARI cases was RSV, with a prevalence rate of 10.65% (70/657), followed by HAdV (7.91%, 52/657) and HIFV (6.09%, 40/657). HMPV was detected in 30 patients (4.57%), HPIV was detected in 24 (3.65%), HBOV was detected in 21 (3.20%), HRV was detected in 11 (1.67%), and HCoV was detected in 3 patients (0.46%).

Table 1 Viral infection profiles in hospitalized patients with SARI in Huzhou, 2017–2019

			Viral infection profiles							
Years	SARI cases	Any viral etiology	HRSV	HAdV	HIFV	HMPV	HPIV	HBOV	HRV	HCoV

2017	191	87	32	18	8	8	10	8	3	0
2018	204	74	17	8	21	14	5	7	2	0
2019	262	90	21	26	11	8	9	6	6	3
~~Totol~~	657	251	70	52	40	30	24	21	11	3

Epidemiology of HAdV

~~During our study period, HAdV was the second common viral pathogen detected in SARI cases, with a detection rate of 7.91% (52/657). As shown in Table 2,~~ among the 52 HAdV-infected patients, 31 (58.33%) were male and 21 (41.67%) were female. No significant difference was observed in males and females in the HAdV-infected cases (P = 0.481). Infection with HAdV was found in all age groups tested(0～, 2～, 5～,15～, 50～, 65～). Children under 15 years old accounted for 84.62% (44/52) of the infections. There were no significant differences in HAdV detection rates among different age groups (P = 0.467). The highest detection rate was in the 2–<5 year age group(9.44%), followed by 5–<15 years (9.13%), 15–<50 years (7.14%), 0–<2 years (5.05%), 50–<65 years (3.13%)and ≥65 years (2.86%).

Table 2 HAdV-positive in hospitalized patients of different ages and gender with SARI

Variable	Tested SARI cases N (percentage)	HAdV-positive cases N (percentage)	HAdV-negtive cases N (percentage)	Positive rate	χ^2	P
~~Gender~~					0.497	0.481
Male	361（53.40）	31（58.33）	330(54.55)	8.59%		
Female	296（46.60）	21（41.67）	275(45.45)	7.09%		
Age (years)					0.431	0.476
0～	99（15.07）	5（9.62）	94（16.55）	5.05%		
2～	180（27.40）	17（32.69）	163（23.51）	9.44%		
5～	241（36.68）	22（42.31）	219（43.05）	9.13%		
15～	70（10.65）	5（9.61）	65（6.95）	7.14%		
50～	32（4.87）	1（1.92）	31（4.64）	3.13%		
65～	35（5.33）	1（1.92）	34（5.30）	2.86%		
Total	657	52	605	7.91%		

Fig. 1 Monthly distribution of HAdV infections from January 2017 to December 2019

HAdV detection rate varied from year to year, from 9.42% (18/191) in 2017, 3.92% (8/204) in 2018 to 9.92% (26/262) in 2019 (Table 1). The monthly distribution of HAdV infections is shown in Fig. 1. HAdV was detected in every month throughout the study period except January. Higher activity of HAdV infection could be seen from spring to early autumn (April to September), and the detection rate in September reached a peak of 28.17%. In contrast, lower activity of HAdV infection were observed during late autumn to winter (from October to February), when the average detection rate was only 2.37%.

Additionally, 13.46–% (n = 7) of the 52 HAdV-infected cases were co-detected with other respiratory pathogens. RSV (n = 3) was the most frequently co-detected virus. HPIV (n = 2), HMPV (n = 1) and HRV (n =1) were also found to be co-infected with HAdV.

HAdV genotyping and phylogenetic analysis

Of the 52 HAdV--positive samples confirmed by real-time RT-PCR, 30 samples were successfully sequenced and genotyped by nested-PCR. Phylogenetic analysis based on partial hexon sequences indicated that 4 species (A, B, C, E) of HAdV, including 7 different types were identified throughout the study period~~, see Fig.2~~. HAdV-B3 (n = 17, 56.67 %) was the most prevalent HAdV types, followed by HAdV-B7 (n = 5, ~~16367~~ %) and HAdV-E4 (n = 3, 10.00 %). HAdV-C1 (n = 2, 6.67 %), HAdV-C2(n =1,3.33 %), HAdV-B21(n =1,3.33 %) and HAdV-B55(n =1,3.33 %)were also detected. The genotype distribution of HAdV infections in each month is shown in Fig. 3. The predominant genotypes of HAdV during our study period varied according to surveillance year. Overall, HAdV-B3 was the most frequently detected genotype in 2017and 2019, accounting for 75.00% (9/12) and 63.64(7/11) of typed HAdV infections ~~in 2017 and 2019~~ respectively. ~~5~~ different types were detected in 2018, including HAdV-B7 (n = 2), HAdV-C1 (n = 2), HAdV-B3 (n = 1), HAdV-B55 (n = 1) and HAdV-C2 (n = 1).No predominant strain was responsible for HAdV infections in 2018, although HAdV-B7 (28.57%, 2/7) and HAdV-C1 (28.57%, 2/7) were the major causative genotypes.

Fig. 2 Phylogenetic analyses based on partial hexon sequences of HAdV strains. The trees were generated using the neighbor-joining method, validated by 1000 bootstrap replicates. Bootstrap values ⩾ 70% are shown on the branch. HAdV sequences identified in this study are indicated by closed circles.

Fig. 3 Distribution of HAdV genotypes detected according to month.

Discussion

SARI is one of the most common diseases in human and the leading cause of hospitalization in children worldwide[15, 16]. Because the early clinical symptoms of respiratory infections caused by viruses are similar, and the imaging findings lack specificity, pathogen detection is very important in clinical diagnosis and epidemiological monitoring. The present study was carried out from January 2017 to December 2019 among hospitalized patients with SARI in Huzhou, China. During the study period, a total of 657 hospitalized SARI cases were enrolled, of which 80.57% were children under 15 years of age. ~~It suggests~~ that SARI is still an important factor affecting the health of local children. 38.20% of hospitalized SARI cases in our study exhibited at least one respiratory virus, which was consistent with pr[illegible]us reports from China (33.44%-41.50%)[17, 18]and other countries (37.57%-41.8%)[19, 20].

HAdV was the second common viral pathogen detected, with a detection rate of 7.08%, which is lower than the finding in SARI cases of hospitalized children in Beijing (11.90%) and Shanghai (14.70%)[21]. Previous studies have indicated that HAdV is the major pathogen that causes respiratory tract infections in children, especially for children younger than 5 years[8, 10]. As expected, we found that HAdV infection mainly occurred in children under 15 years of age (84.62%), and the detection rate reached a peak (9.44%) in children aged 2 to ≤5 years.

Previous studies have shown that the epidemic peak seasons of HAdV-associated respiratory infections varies in different parts of China, and even in different monitoring years in the same region. Our study revealed that HAdV showed higher activity in the relatively high temperature seasons (spring to early autumn), which is similar to what has been found in Beijing (Northern

China)[8]and Guangzhou(Southern China)[13], where HAdV infections occurred throughout the year with the highest prevalence in the summer. However, this finding is discordant with other studies conducted in Northe[illegible]hina that have reported seasonal peaks for HAdV infections in winter and spring[9, 12]. It is worth mentioning that the surveillance period of the above-mentioned studies conducted in different regions of China varies, and the predominant HAdV types circulated are also different. A recent study from Hunan indicates that different HAdV types showed a different seasonal distribution patterns: HAdV-3 was the predominant type of HAdV infection during summer, while HAdV-7 had the highest detection rate during spring[11]. Based on the above research, we speculate that the discrepant seasonal peak for HAdV infections are not only related to regional differences, but also related to the major types of HAdV circulating locally.

Globally the types most commonly associated with respiratory syndromes belong to HAdV species B, C or E. Many studies have reported that HAdV–B3, HAdV–B7 and HAdV–C2 are the most prevalent types in China, but the predominant type distribution vary among different regions and change over time. For example, most of HAdV-positive cases were caused by HAdV-B3 from 2012 to 2013 in Southern China[13], while HAdV-B7 dominated in Northern China during the same study period[10]. However, recent reports indicated that the most predominant types have changed into HAdV-B3 and HAdV-C2 in some Northern cities of China in 2017-2018[8, 9]. Throughout the present study period, 7 different types of HAdV belonging to 4 species (HAdV-A, B, C, E) were identified in hospitalized SARI cases, with HAdV-B3 as the most prevalent HAdV types, followed by HAdV-B7 and HAdV-E4. Our monitoring data showed that no type of HAdV presented absolutely predominant during HAdV epidemic seasons, HAdV-B3 was co-circulating with other types and presented an alternate prevalence pattern. Overall, HAdV-B3 was the most frequently detected genotype in 2017. No predominant strain was responsible for HAdV infections in 2018, with HAdV-B7 and HAdV-C1 as the major causative genotypes. HAdV-B3 re-emerged as the predominant genotype in 2019. Similar epidemic pattern were observed in a prolonged surveillance study conducted in southeastern China, where HAdV-7 and HAdV-3 alternate as the predominant genotypes causing pediatric pneumonia[22]. It is worth noting that in 2017 and 2019, when HAdV-3 presented as the predominant type detected, the ~~detected~~ rate of HAdV was significantly higher than that in 2017(9.42% in 2017, 3.92% in 2018 and 9.92%in 2019). The reasons need to be further explored. During HAdV infection, neutralizing antibodies are formed against the epitopes located in the hyper variable regions (HVRs) of the hexon protein. Just recently, Haque E et al. ~~explore~~ the variation in HVRs of hexon among globally distributed strains of HAdV-3[23]. They found that the HVRs of HAdV-3 strains circulating worldwide were highly heterogeneous and have been mutating continuously since -their original isolation and suggested that~~,~~ this heterogeneity may explain the worldwide increased prevalence of HAdV-3 respiratory infections.

Recent HAdV epidemiology studies showed that there was very high co-infection rate between HAdV and other pathogens in respiratory tract infection cases (37.50%-74.85%)[8, 9, 11]. In our study, coinfection of HAdVs and other respiratory viruses was only detected in 13.46 %of the SARI cases. Such discrepant co-infection rate may be caused by the different selection criteria of the research objects and methodological differences.

Our study is limited by a single-site setting, small sample size, and especially the partial genotyping of detected HAdVs. Genotyping was only successful for 57.69% (30/52) of HAdV

infection cases. Besides, typing of HAdV was merely performed by sequencing of partial hexon gene in the present study, which is hard to find any potential recombination between different types of HAdV strains.

Conclusions

In conclusion, this study revealed the prevalence and molecular epidemiological characteristics of HAdV infections among hospitalized patients with SARI in Huzhou from January 2017 to December 2019. HAdV was the second common viral pathogen detected in SARI cases, with most (84.62%) HAdV-positives cases detected among children < 15 years of age. Higher activity of HAdV infection could be seen in spring -early autumn season. No type of HAdV presented absolutely predominant during HAdV epidemic seasons, HAdV-B3 was co-circulating with other types and presented an alternate prevalence pattern. Our results provide a reliable scientific basis to better understand the role played by HAdVs in SARI cases, and for the prevention and control of HAdV infection.

Authors' contributions

LJ wrote the first draft and did the phylogenetic analysis. XFW and DSX participated in the HAdVs detection. LPC participated in the Genomic amplification for genotyping. GTL did the epidemiological investigation and performed the statistical analysis. All authors read and approved the final manuscript.

Acknowledgements

We thank the staff of the First People's Hospital in Huzhou for collecting the samples.

References

1. Hoeben RC, Uil TG. Adenovirus DNA replication. Cold Spring Harb Perspect Biol. 2013;5(3).
2. Ghebremedhin B. Human adenovirus: Viral pathogen with increasing importance. Eur J Microbiol Immunol. 2014;4(1):26-33.
3. Lynch JP, 3rd, Kajon AE. Adenovirus: Epidemiology, Global Spread of Novel Serotypes, and Advances in Treatment and Prevention. Semin Respir Crit Care Med. 2016;37(4):586-602.
4. Sandkovsky U, Vargas L, Florescu DF. Adenovirus: current epidemiology and emerging approaches to prevention and treatment. Curr Infect Dis Rep. 2014;16(8):014-0416.
5. Lee J, Choi EH, Lee HJ. Comprehensive serotyping and epidemiology of human adenovirus isolated from the respiratory tract of Korean children over 17 consecutive years (1991-2007). J Med Virol. 2010;82(4):624-31.
6. Lin MR, Yang SL, Gong YN, Kuo CC, Chiu CH, Chen CJ, et al. Clinical and molecular features of adenovirus type 2, 3, and 7 infections in children in an outbreak in Taiwan, 2011. Clin Microbiol Infect. 2017;23(2):110-6.
7. Zhu Z, Zhang Y, Xu S, Yu P, Tian X, Wang L, et al. Outbreak of acute respiratory disease in China caused by B2 species of adenovirus type 11. J Clin Microbiol. 2009;47(3):697-703.
8. Yao LH, Wang C, Wei TL, Wang H, Ma FL, Zheng LS. Human adenovirus among hospitalized children with respiratory tract infections in Beijing, China, 2017-2018. Virol J. 2019;16(1):019-1185.
9. Zhao MC, Guo YH, Qiu FZ, Wang L, Yang S, Feng ZS, et al. Molecular and clinical characterization of human adenovirus associated with acute respiratory tract infection in hospitalized children. J Clin Virol. 2020;123(104254):25.

10. Liu C, Xiao Y, Zhang J, Ren L, Li J, Xie Z, et al. Adenovirus infection in children with acute lower respiratory tract infections in Beijing, China, 2007 to 2012. BMC Infect Dis. 2015;15(408):015-1126.

11. Xie L, Zhang B, Xiao N, Zhang F, Zhao X, Liu Q, et al. Epidemiology of human adenovirus infection in children hospitalized with lower respiratory tract infections in Hunan, China. J Med Virol. 2019;91(3):392-400.

12. Li Y, Zhou W, Zhao Y, Wang Y, Xie Z, Lou Y, et al. Molecular typing and epidemiology profiles of human adenovirus infection among paediatric patients with severe acute respiratory infection in China. PLoS One. 2015;10(4).

13. Chen Y, Liu F, Wang C, Zhao M, Deng L, Zhong J, et al. Molecular Identification and Epidemiological Features of Human Adenoviruses Associated with Acute Respiratory Infections in Hospitalized Children in Southern China, 2012-2013. PLoS One. 2016;11(5).

14. Lu X, Erdman DD. Molecular typing of human adenoviruses by PCR and sequencing of a partial region of the hexon gene. Arch Virol. 2006;151(8):1587-602.

15. Williams BG, Gouws E, Boschi-Pinto C, Bryce J, Dye C. Estimates of world-wide distribution of child deaths from acute respiratory infections. Lancet Infect Dis. 2002;2(1):25-32.

16. Chakhunashvili G, Wagner AL, Power LE, Janusz CB, Machablishvili A, Karseladze I, et al. Severe Acute Respiratory Infection (SARI) sentinel surveillance in the country of Georgia, 2015-2017. PLoS One. 2018;13(7).

17. Zheng YX, Chen J, Kong DC, Pan H, Zhou YQ, Chen ML, et al. [Pathogenic characteristics of hospitalized severe acute respiratory infections in Shanghai, China, 2015-2017]. Zhonghua Liu Xing Bing Xue Za Zhi. 2019;40(8):911-6.

18. Xu W, Guo L, Dong X, Li X, Zhou P, Ni Q, et al. Detection of Viruses and Mycoplasma pneumoniae in Hospitalized Patients with Severe Acute Respiratory Infection in Northern China, 2015-2016. Jpn J Infect Dis. 2018;71(2):134-9.

19. Chittaganpitch M, Waicharoen S, Yingyong T, Praphasiri P, Sangkitporn S, Olsen SJ, et al. Viral etiologies of influenza-like illness and severe acute respiratory infections in Thailand. Influenza Other Respir Viruses. 2018;12(4):482-9.

20. Alroy KA, Do TT, Tran PD, Dang TQ, Vu LN, Le NTH, et al. Expanding severe acute respiratory infection (SARI) surveillance beyond influenza: The process and data from 1 year of implementation in Vietnam. Influenza Other Respir Viruses. 2018;12(5):632-42.

21. Zhao Y, Lu R, Shen J, Xie Z, Liu G, Tan W. Comparison of viral and epidemiological profiles of hospitalized children with severe acute respiratory infection in Beijing and Shanghai, China. BMC Infect Dis. 2019;19(1):019-4385.

22. Wo Y, Lu QB, Huang DD, Li XK, Guo CT, Wang HY, et al. Epidemical features of HAdV-3 and HAdV-7 in pediatric pneumonia in Chongqing, China. Arch Virol. 2015;160(3):633-8.

23. Haque E, Banik U, Monowar T, Anthony L, Adhikary AK. Worldwide increased prevalence of human adenovirus type 3 (HAdV-3) respiratory infections is well correlated with heterogeneous hypervariable regions (HVRs) of hexon. PLoS One. 2018;13(3).

Figure1

Click here to access/download;Figure;figure1.pdf

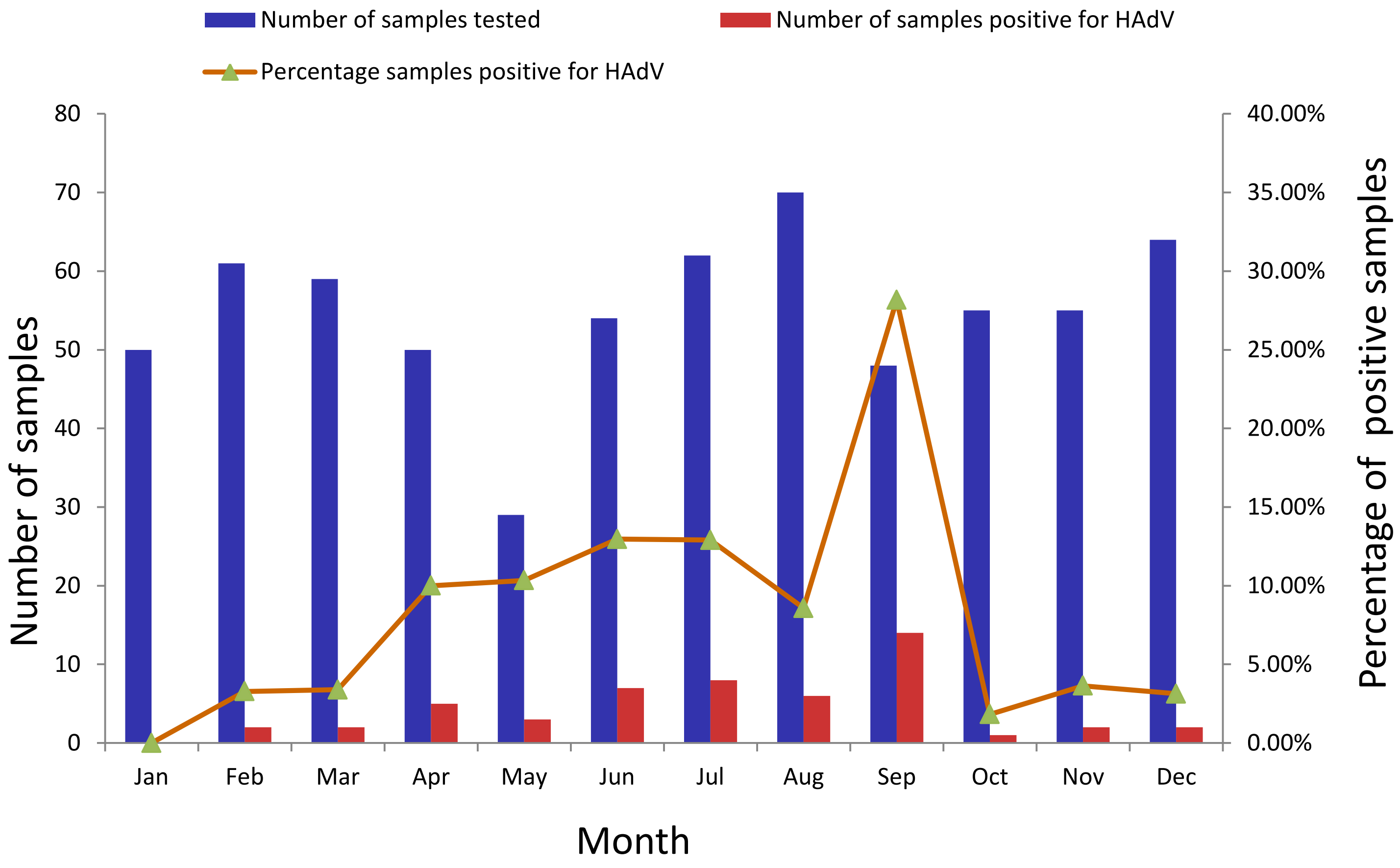

Figure2

Click here to access/download;Figure;figure2.pdf

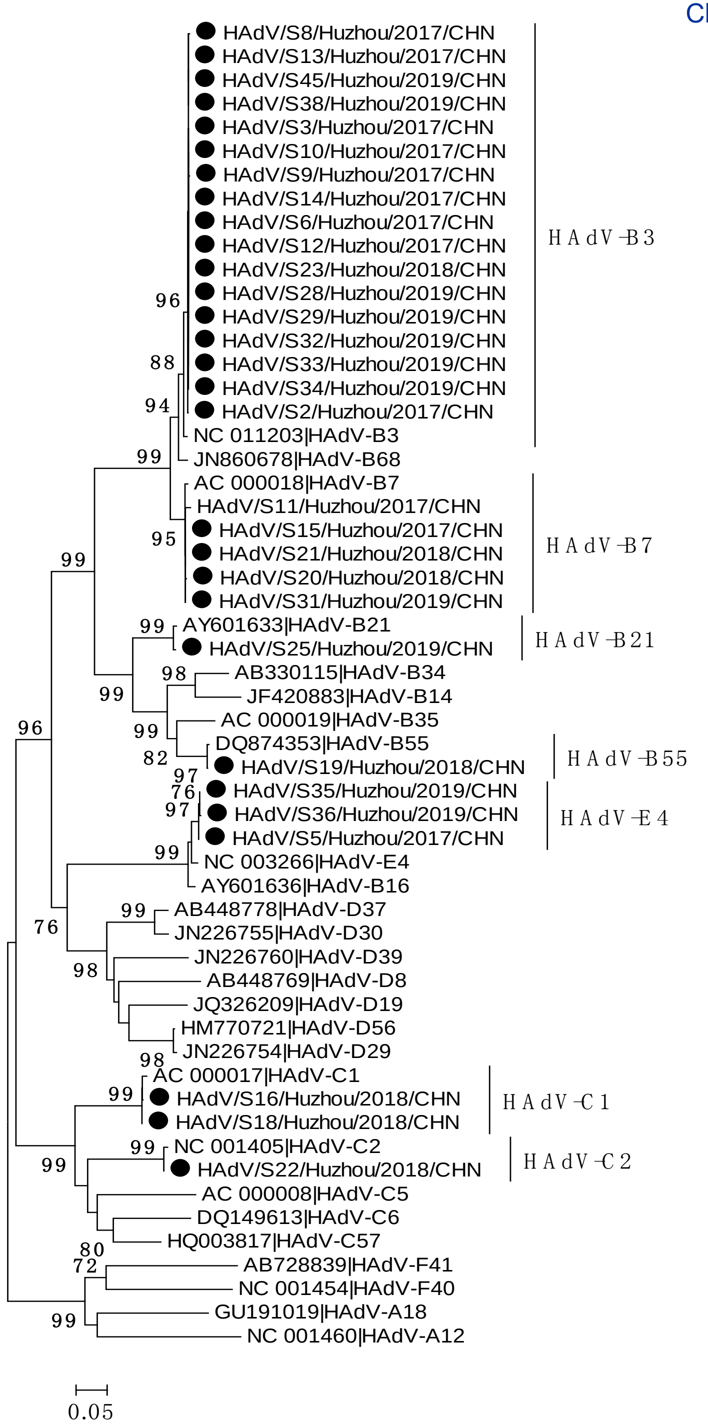

Figure3

Click here to access/download;Figure;figure3.pdf

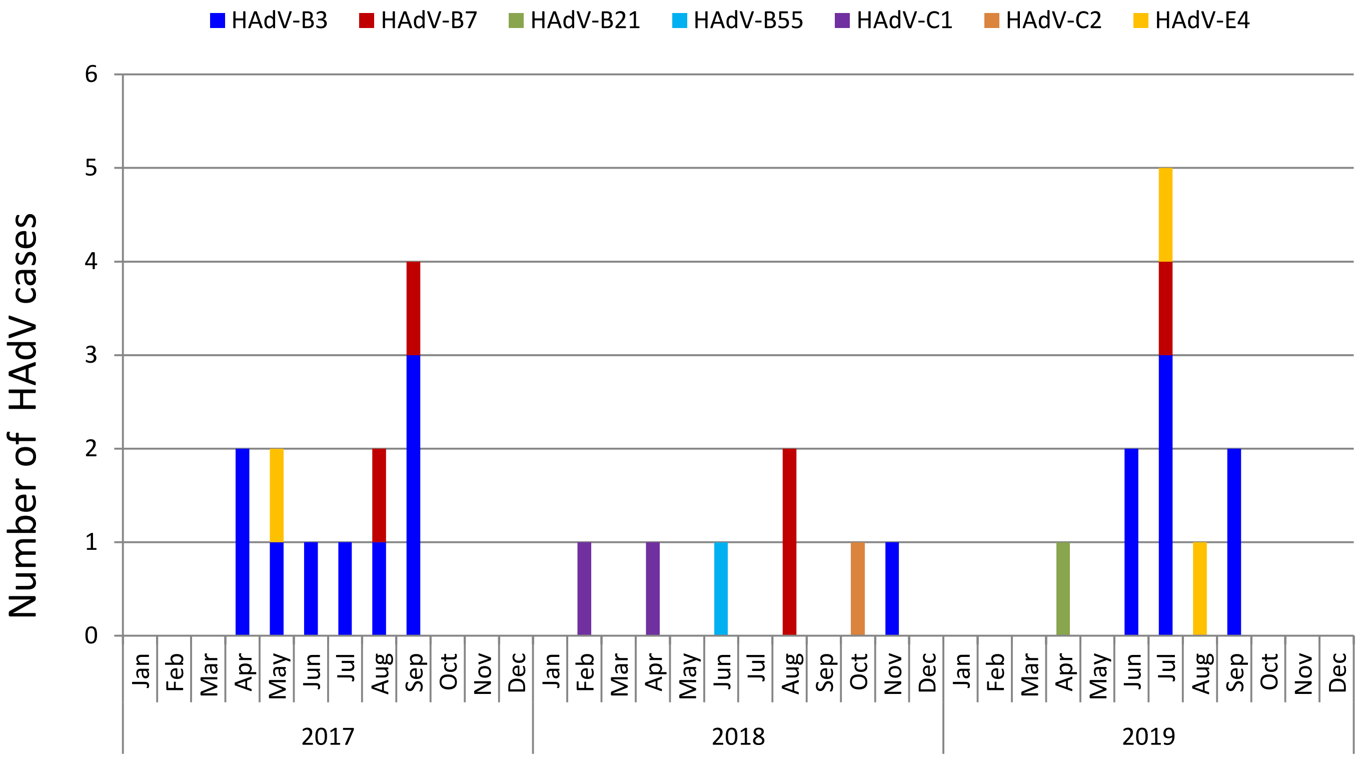

Supplement: Attachment — Submitted filename: PONE-D-21-05692_reviewer.pdf [file pone.0265987.s002.pdf]
