# Supplementary material for: Molecular typing and epidemiology profiles of human adenovirus infection among hospitalized patients with severe acute respiratory infection in Huzhou, China
Source: PLoS One. 2022 Apr 21;17(4):e0265987. doi: 10.1371/journal.pone.0265987 (PMC9022850; doi:10.1371/journal.pone.0265987)
Supplement: Attachment — Submitted filename: Response to Reviewers.docx [file pone.0265987.s003.docx]

**Rebuttal letter**

Dear Editor Rosa Maria Wong-Chew,

Thank you very much for giving us an opportunity to revise our manuscript. We also appreciate reviewers very much for their positive and constructive comments and suggestions on our manuscript entitled “Molecular Typing and Epidemiology Profiles of Human Adenovirus Infection among Hospitalized Patients with Severe Acute Respiratory Infection in Huzhou, China” (PONE-D-21-05692).

We have carefully addressed all of the comments from the reviewers, as outlined in the point-by-point responses attached below. We hope that you find the revised manuscript now acceptable for publication in PLOS ONE.

Updated statement: This work was supported by grants from Natural Science Foundation of Huzhou Science and Technology Bureau (grant number: 2021YZ23), the funders had no role in study design, data collection and analysis, decision to publish, or preparation of the manuscript.

Yours Sincerely

Lei Ji

**Responses to the reviewers’ comments:**

(Q as comments, A as our responses)

**Reviewer 1**
**Q:** Considering that HAdV is an important respiratory virus worldwide, I suggest discussing the findings of the study with studies performed in other countries, such as in Europe (Price RHM et al., https://doi.org/10.1038/s41598-018-37481-y) and South America (Pscheidt et al., DOI: 10.1002/rmv.2189). When discussing HAdV seasonality, I suggest comparing the study with those from other geographic regions, as viral circulation has been associated with temperature and circulation of other respiratory viruses.

**A:** Thanks for the reviewer’s suggestion. The two references mentioned above have been added (see line 230, 239,242). “Most respiratory viral infections have seasonality, of note, this seasonality might vary according to geographical location. Price RHM et al. have investigated the relationship between meteorological factors and viral seasonality in Scotland over a 6.5-year period. In their study, HAdV is present throughout the year without a clear seasonality and prefer temperatures around 9 °C. In another study conducted in patients with respiratory infection in southern Brazil, HAdV circulated year‐round, with higher frequency during winter and early spring.” We have added these sentences to the manuscript, see line 238-245.

**Q:** There are several English and typing details that should be revised and corrected, as pointed in the pdf file. For example, symbols such as ~ to describe age group (0～, 2～, 5～,15～, 50～, 65～) does not make much sense. Please use 0≤2; 2≤5; 5≤15, etc.

**A:** Thanks for the reviewer’s suggestion. Corrections have been made in the revised version.

**Q:** In the methodology, I believe there is a step missing in the PCR description. Isn't there an extension step at 72ºC after the annealing step?

**A:** According to the multiplex real-time PCR kit’s instructions, the qPCR cycling program was as 50 °C for 10 min, 95 °C for 5min, followed by 40 cycles of 95 °C for 10 s, and 55 °C for 40 s. The same temperature (55 °C) is used for the annealing and extension steps.

**Q:** In Table 1, I suggest including the percentage in addition to the number of cases – N (%).

In Table 2, use Sex instead of Gender.

**A:** Thanks for the reviewer’s suggestion. Corrections have been made in the revised version .

**Q:** Other comments and suggestions can be found in the pdf file (attached).

**A:** Thanks. Corrections have been made in the revised version according to comments and suggestions in the pdf file.

**Reviewer 2**
**Q:** I have only one comment: The authors were unable to type up to 42.3% (22/52) of the study samples. What could explain this high failure rate.

**A:** Thanks for the reviewer’s suggestion. Since the sensitivity of ordinary RT-PCR is lower than that of real-time RT-PCR, more accurate genotyping was only possible for 57.7% (30/52) of the HAdV-positive samples confirmed by real-time RT-PCR, the remaining 22 samples with most of them got a cycle threshold (Ct) ≥ 30 were failed to genotyped.
