# Supplementary material for: Molecular typing and epidemiology profiles of human adenovirus infection among hospitalized patients with severe acute respiratory infection in Huzhou, China
Source: PLoS One. 2022 Apr 21;17(4):e0265987. doi: 10.1371/journal.pone.0265987 (PMC9022850; doi:10.1371/journal.pone.0265987)
Supplement: Attachment — Submitted filename: Response to Reviewers.docx [file pone.0265987.s005.docx]

**Rebuttal letter**

Dear Editor Jianhong Zhou,

Thank you very much for giving us an opportunity to revise our manuscript. We also appreciate reviewers very much for their positive and constructive comments and suggestions on our manuscript entitled “Molecular Typing and Epidemiology Profiles of Human Adenovirus Infection among Hospitalized Patients with Severe Acute Respiratory Infection in Huzhou, China” (PONE-D-21-05692).

We have carefully addressed all of the comments from the reviewers, as outlined in the point-by-point responses attached below. We hope that you find the revised manuscript now acceptable for publication in PLOS ONE.

Updated statement: This work was supported by grants from Natural Science Foundation of Huzhou Science and Technology Bureau (grant number: 2021YZ23), the funders had no role in study design, data collection and analysis, decision to publish, or preparation of the manuscript.

Yours Sincerely

Lei Ji

**Responses to the reviewers’ comments:**

(Q as comments, A as our responses)

**Reviewer 1**
**Q:** for age groups 0≤2, 2≤5, 5≤15 etc. means that 2 years old are included in both groups 0-2 and 2-5. The correct is 0<2, 2<5, 5<15 and so on.

**A:** Thanks for the reviewer’s suggestion. Corrections have been made in the revised version.

**Q:** With regards to HAdV in southern Brazil, it is more frequent during the winter, however, in relation to other respiratory viruses, HAdV is more prevalent in summer

**A:** Thanks for the reviewer’s suggestion. Corrections have been made in the revised version, see line 237-241.

**Q:** Other comments and suggestions can be found in the pdf file (attached).

**A:** Thanks. Corrections have been made in the revised version according to comments and suggestions in the pdf file.
